# Negeviruses isolated from mosquitoes in the Brazilian Amazon

**DOI:** 10.1186/s12985-022-01743-z

**Published:** 2022-01-21

**Authors:** Ana Cláudia da Silva Ribeiro, Lívia Caricio Martins, Sandro Patroca da Silva, Daniele Barbosa de Almeida Medeiros, Keissy Karoline Pinheiro Miranda, Joaquim Pinto Nunes Neto, Hamilton Antônio de Oliveira Monteiro, Bruna Lais Sena do Nascimento, Jose Wilson Rosa Junior, Ana Cecilia Ribeiro Cruz, Pedro Fernando da Costa Vasconcelos, Valéria Lima Carvalho, Sueli Guerreiro Rodrigues

**Affiliations:** Department of Arbovirology and Hemorrhagic Fevers and Postgraduate Program in Virology, Evandro Chagas Institute, Rodovia BR-316 km 7 s/n, Levilândia, Ananindeua, Pará 67030-000 Brazil

**Keywords:** Arboviruses, Insect-specific viruses, Negevirus

## Abstract

**Background:**

There are several groups of viruses including Insect Specific Viruses (ISV) such as the taxon *Negevirus*, a group of viruses phylogenetically related to plant viruses. Negeviruses replicate in mosquito cells, but not in vertebrate cells.

**Methods:**

Pools of hematophagous arthropods were inoculated in Vero and C6/36 cells. The cells were observed to detect possible cytopathic effect. Then, indirect immunofluorescence, RT-PCR, and nucleotide sequencing were performed.

**Results:**

Seven samples which presented negative results for flaviviruses, alphaviruses and bunyaviruses, but showed cytopathic effect in C6/36 cells were sequenced. We identified the occurrence of a variety of ISVs, most of them belonging to the taxon *Negevirus*: The Brejeira, Negev, Cordoba and Wallerfield viruses, including a new virus for science, tentatively named Feitosa virus.

**Conclusions:**

We detected negeviruses in the Amazon region, including two viruses that were isolated for the first time in Brazil: Cordoba virus and the Negev virus and, a new virus for science: the Feitosa virus.

## Introduction

Insect-specific viruses (ISVs) are viruses that naturally infect mosquitoes and replicate in mosquito cells in vitro, but they do not replicate in vertebrate cells. In recent years, there has been an increase in research on ISVs, and they are increasingly attracting the interest of the scientific community due to the evolution of molecular techniques. [1] Most ISVs are made up of RNA and are distributed in several virus families, such as the families *Peribunyaviridae, Flaviviridae, Reoviridae, Rhabdoviridae, Togaviridae, Mesoniviridae,* and the taxon *Negevirus*. [2]

The taxon *Negevirus* consists of positive-sense single-stranded RNA viruses that have been isolated in many regions around the world, including the Americas, Europe, Africa, and Asia [2–6]. This taxon is classified into two main clades, namely: *Nelorpivirus* and *Sandewavirus* [3].

These viruses have a spherical particle size of 45–55 nm in diameter [7] Most of these viruses consist of three open reading frames (ORFs) flanked by untranslated regions (UTRs) at the 5′ and 3′ ends, while each ORF is separated by short intergenic regions. One large ORF (ORF1) was found to be 233 to 7339 nt and encodes the viral polymerase protein. ORF 2 (medium) and ORF3 (small) encode glycoproteins and membrane proteins, respectively [7, 8]. The large ORF contains putative protein domains that correspond to non-structural proteins. In addition, four functional domains were also identified: (i) a methyltransferase domain at 522 to 1386 nt; (ii) a RNA ribosome methyltransferase domain at the position of 2511 to 3072 nt; (iii) a helicase domain from 4182 to 4908 nt; and (iv) a RNA-dependent RNA-polymerase (RdRp) domain from 5802 to 6927 nt [7].

The negeviruses that have been isolated so far belonging to the genus *Nelorpivirus* are: Big Cypress virus (BCPV), Brejeira virus (BRJV), Corboda virus (CDBV), Loreto virus (LORV), Negev virus (NEGV), Ngewotan virus (NWTV), Piura virus (PIUV), and San Bernardo virus (SBDV). Regarding the *Sandewavirus* genus, the Biratnagar virus (BIRV), Dezidougou virus (DEZV), Goutanap virus (GANV), Santana virus (SANV), Tanay virus (TANAV), and Wallerfield virus (WALV) and Bustos virus (BUSV) were identified [3, 8, 9].

In the current study we describe the genomic characterization of negeviruses isolated from mosquitoes resulted from arbovirus surveillance actions in the Brazilian Amazon, including viruses reported for the first time in Brazil and also a noval negevirus.

## Materials and methods

### Mosquitoes collection methods

The mosquitoes were collected in the areas of Marabá, Curionópolis and Canaã dos Carajás, in the state of Pará, Brazil, in 2014 and 2015. Hematophagous arthropods were collected on the ground and in the canopy of trees using two methods: human attraction protected and enlightened during the day, using hand nets (polyester net bag 30 cm in diameter, attached to a 30 cm aluminum handle) and an oral suction device, which stored captured mosquitoes. The other technique used was light attraction, using CDC light trap (John W. Hock Company, Gainesville, FL, USA) during the night (from 6 PM to 6 AM). After taxonomic identification, arthropods were organized in pools.

### General methodological flow

All mosquitoes samples were inoculated in cells aiming for the virus isolation. Cytopathic effect (CPE) was investigated and registered. Then, all the inoculated cells were submitted to indirect immunofluorescence test to detect *Alphavirus*, *Flavivirus*, *Orthobunyavirus* and *Phlebovirus*; furthermore, we also performed RT-PCR for detection of *Alphavirus*, *Flavivirus* and *Orthobunyavirus*. The culture supernatant presenting CPE had their genome sequenced.

### Virus isolation in cell cultures and indirect immunofluorescence test (IFA)

The *Aedes albopictus* clone C6/36 cell line (ATCC: CRL 1660) [10], and the Vero cell line, originating from African green monkey, *Chlorocebus sabaeus* (ATCC: CCL-81) [11], were maintained in laboratory through cell splitting on a weekly basis. For C6/36 cells (maintained at a temperature of 28 °C), the Leibowitz L-15 medium with L-glutamine (Gibco, MA, USA), supplemented with 5% fetal bovine serum (FBS) (Gibco, MA, USA), tryptose phosphate (Himedia, Mumbai, India) (2.95%), antibiotics (penicillin 10,000 U/L and streptomycin 10,000 g/L) (Gibco, MA, USA) and non-essential amino acids (10 mL/L) (Baktron Microbiology, RJ, Brazil) was used. [12] The Vero cell line, maintained in an incubator at 37 °C and 5% CO_2_ (Thermo Scientific, MA, USA) was weekly splitted using a solution of trypsin (0.25%) (Difco, NJ, USA) with ethylenediaminetetraacetic acid (EDTA) (Invitrogen, MA, USA) for cell dissociation. The Vero cells were maintained in medium 199 (Gibco, MA, USA) supplemented with sodium bicarbonate 2.2 g/L (Sigma, NJ, USA), 5% of FBS and antibiotics (penicillin 10,000 U/L and streptomycin 10,000 g/L).

For processing the hematophagous arthropods, suspension of mosquitoes was prepared in 2 mL eppendorf-type tube (U-bottom) with 1 mL of D-PBS 1 × diluent (Gibco, MA, USA) with 2% penicillin and streptomycin, 1% fungizone, and 5% FBS. Then, grinding of the mosquitoes was performed in the Tissuelyser equipment for 60 s using a 3 mm tungsten bead (frequency: 25 Hz). After grinding, the tubes with the arthropods were frozen in a freezer at − 80 °C overnight, and, the next day or at the moment of inoculation, thawed and centrifuged in a refrigerated centrifuge at 4 °C at 11,200*g* for 10 min [13, 14].

Cells were inoculated from one to three days after cell splitting (confluence of approximately 90%). Immediately before inoculation, the growth medium (5% FBS) was discarded from the 24-well plates containing the cells. The C6/36 and Vero cell cultures were concomitantly inoculated with the specimen’s suspension. Each well of the plate was inoculated with 100 µl of the supernatant from a batch of arthropods, and each plate included positive controls, cells inoculated with arboviruses that replicate in these cells, such as Chikungunya virus (CHIKV), Dengue virus (DENV) and/or Oropouche virus (OROV), and a negative control (uninoculated cells). After inoculation, the plates were incubated in an incubator for one hour at 28 °C (C6/36 cells) or 37 °C (Vero cells), and shaken gently every 15 min. Subsequently, 1.5 mL of L-15 maintenance medium was added to each well of the plate with C6/36 cells and 1.5 mL of 199 maintenance medium for Vero cells. The plates were then observed on a daily basis for seven days under an inverted microscope to visualize possible cytopathic effect and possible other abnormalities in the cell monolayer [15, 16].

To confirm cell infection and identify the viral agent, the indirect immunofluorescence test (IFA) was performed according to the protocol adapted from Gubler et al. [17]. The cell suspensions were tested using mouse polyclonal antibodies (produced in house) of group of arboviruses included at the genera *Alphavirus*, *Flavivirus*, *Orthobunyavirus* and *Phlebovirus*.

### RT-PCR for *Alphavirus*, *Flavivirus* and *Orthobunyavirus* detection

RT-PCR was performed for *Flavivirus, Alphavirus* and *Orthobunyavirus* detection. RNA extraction of cell supernatant was performed using the Maxwell 16 equipment with the Maxwell 16 Total RNA purification kit (Promega, WI, USA) according to the manufacturer's instructions.

After extraction, total RNA was reverse transcribed with 12,5 ng/µl random hexamers (Invitrogen, MA, USA), 0,5 mM dNTPs, ultrapure water and denatured at 65 °C for 5 min. 5 × buffer, 5mMDTT, 40U RNAse inhibitor (RNAse OUT, Invitrogen, MA, USA) and 200U reverse transcriptase (SuperScript III, Invitrogen, MA, USA or M-MLv Invitrogen, MA, USA) were then added to the mix, which was then incubated at 25 °C for 5 min. For flaviviruses (220 bp), we used the ‘forward primer cFD2’ (GTGTCCCAGCCGGCGGTGTCATCAGC) and the ‘reverse MA’ (CATGATGGGRAARAGRGARRAG); for alphaviruses (434 bp), the ‘forward primer MAY 1’ (YAGAGCDTTTTCGCAYSTRGCHW) and ‘reverse MAY 2’ (ACATRAANKGNGTNGTRTCRAANCCDAYCC) were used; and for orthobunyavirus, (300 bp), the ‘forward primer BUN-S’ (AGTAGTGTGCTCCAC) and ‘reverse BUN-C’ (AGTAGTATACTCCAC) were used.

The cDNA synthesis was carried out at 55 °C for one hour, followed by 70 °C for 15 min and kept at 4 °C until the PCR step. PCR amplification was performed with 5 μL of cDNA mixed with 10 × buffer, 1.5 mM MgCl_2_, 0,2 mM dNTP, 0.2 µM forward primer, 0.2 µM reverse primer, 2U Taq DNA polymerase (Invitrogen, MA, USA) and ultrapure water to 50 μL reaction volume. The cycle conditions are described in the Table 1.

**Table 1:** Thermocycling programs used for each pair of genera universal primers

Genera	No. of cycles	Denaturation	Hybridization/increment	Elongation	Final elongation
*Flavivirus*	35	94 °C por 2′	94 °C por 30″ 55 °C por 30″	72 °C por 2,5′	72 °C por 5′
*Alphavirus*	45	95 °C por 5′	95 °C por 30″ 55 °C por 30″	68 °C por 1′	68 °C por 5′
*Orthobunyavirus*	35	94 °C por 5′	94 °C por 1′ 55 °C por 1′	72 °C por 2′	72 °C por 7′

PCR products were revealed by using 3% agarose gel electrophoresis (Ultra pure gel—Invitrogen, MA, USA) in 1X T.A.E. buffer (10 mM Tris; 0.1 M Acetate; 1 mM EDTA pH 7.2) stained with SYBR® Safe DNA gel stain (Invitrogen, MA, USA), in a 50-min run using a transilluminator with an ultraviolet light source [18].

### Nucleotide sequencing

For RNA extraction, the commercial QIAamp®Viral RNA Mini Kit was used, following the manufacturer's recommendations. Sequencing first occurred with reverse transcription and the second cDNA strand was obtained with the cDNA Synthesis System kit (Roche Diagnostics, Basel, Switzerland) by using random primers (400 μM Roche "random" Primer). The product of this reaction was purified by using the magnetic beads from the Agencourt AMPure XP Reagent kit (Beckman Coulter, CA, USA). The transcribed and purified product served as input for genomic library preparation by applying the methodology described in the Nextera XT DNA Library Preparation Kit.

The library was evaluated in terms of quantity through the Qubit® 2.0 Fluorometer and the Qubit® dsDNA HS Assay Kits, and in terms of the size of the fragments produced, by using the High Sensitivity DNA Analysis Kits (Agilent Technologies, CA, USA) and the Bioanalyzer 2100 (Agilent Technologies, CA, USA). After the library checking steps, sequencing was performed by using the synthesis methodology via the MiniSeq platform (Illumina, CA, USA) using the MiniSeq High Output kit (300 cycles), according to the manufacturer's instructions.

The generated files were treated and used for assembly by using the same methodology using SPAdes v.3.13.0 [19] and IDBA-UD v.1.1.3 [20]. The k-mer values for SPAdes of 21, 33, 55, 77 and for IDBA-UD the program's default k-mer were used. Initially, a Multiple Sequencing Alignment (MSA), using the entire ORFs of the Brazilian strain and sequences available on NCBI, was performed using Mafft v7.310 software [21]. The aligned data was statistically evaluated to identify the best amino acid replacement model to be applied in the phylogenetic analysis by using the ProtTest v.3 software [22]. Subsequently, the Maximum Likelihood methodology was employed through the RaxML (Randomized Axelerated Maximum Likelihood) software, which is used to build the phylogenetic tree [23]. The bootstrap test was also applied along with the maximum-likelihood estimation (MLE) method by fixing 1000 replicates to provide reliability to the values of the clusters [24].

## Results

**Fig. 1:**
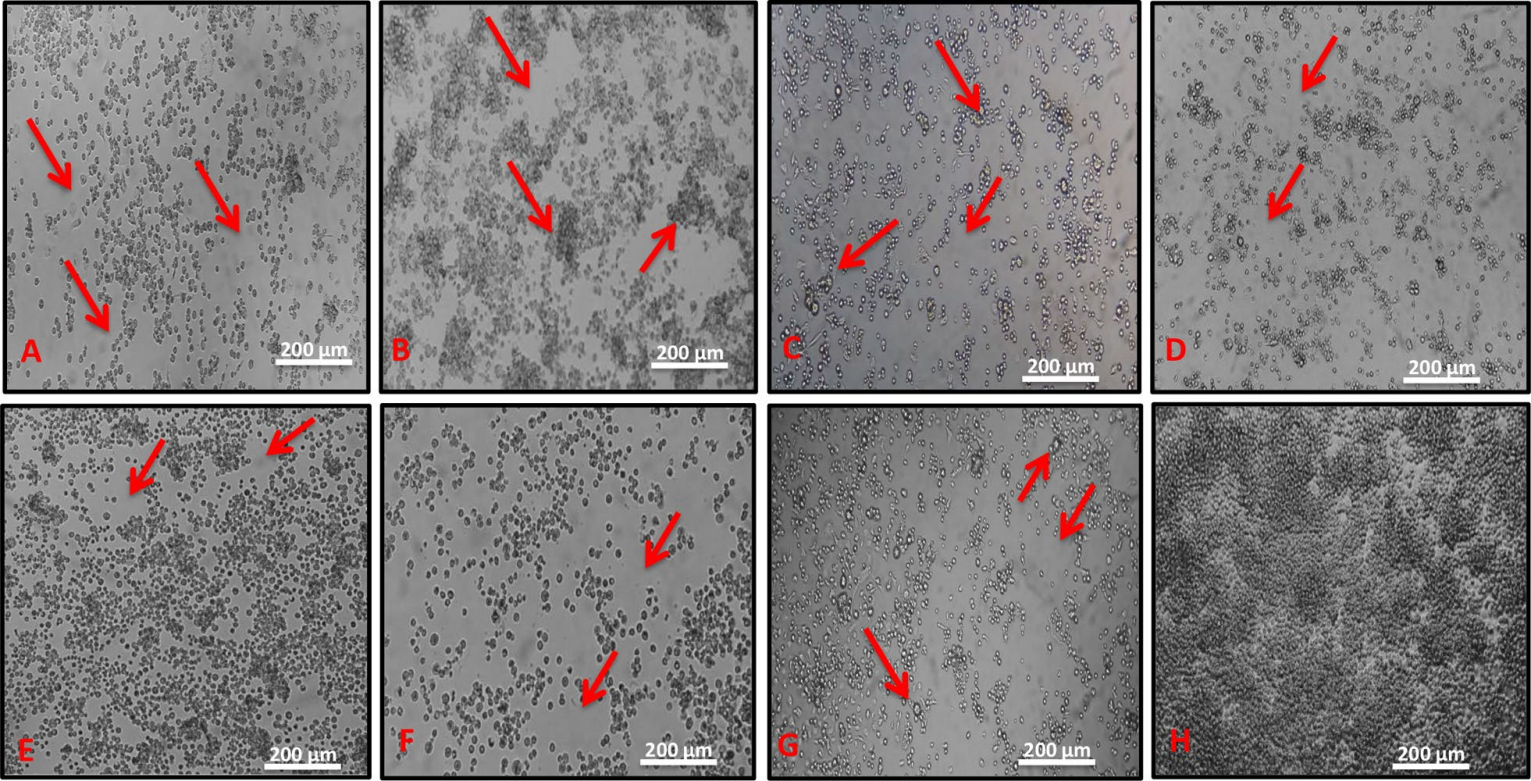
Cytopathic effect (CPE) observed in infected C6/36 cells (red arrow). **a** Cells inoculated with the sample BE AR 805503 (CPE at 6th day post-infection), showing destruction of cell’s monolayer; **b** cells inoculated with the sample BE AR 805529 (CPE at 4th day post-infection), showing destruction of cell’s monolayer and formation of clumps; **c** cells inoculated with the sample BE AR 805511 (CPE at 6th day post-infection), showing destruction of cell’s monolayer and large cells (larger than normal cells); **d–f** cells inoculated with the sample BE AR 805514 (CPE at 6th day post-infection), BE AR 820396 (CPE at 6th dpi) and BE AR 805520 (CPE at 6th day post-infection), respectively, showing destruction of the cell’s monolayer; **g** cells inoculated with the sample BE AR 805525 (CPE at 6th day post-infection), highlighting destruction of the cell’s monolayer and large cells (larger than normal cells); **h** negative control (C6/36 cells) showing no CPE (100X)

The samples studied were negative for *Flavivirus, Alphavirus*, *Orthobunyavirus* and *Phlebovirus* through IFA and also negative by RT-PCR for three viral genera (*Flavivirus, Alphavirus* and *Orthobunyavirus*). Although, seven pools of mosquitoes (BE AR 805503, BE AR 805511, BE AR 805514, BE AR 805520, BE AR 805525, BE AR 820396, BE AR 805529) presented CPE in these cells, but not in Vero cells. The isolates presented lytic-type CPE in C6/36 cells, characterized by the presence of dead cells as well as refringent cell, clumps and syncytial formation until complete destruction of the single layer between day four and day six after inoculation (Fig. 1).

No arbovirus genome was detected through sequencing, while sequences of 14 strains of ISVs belonging to the taxon *Negevirus* were obtained: BRJV (6 strains), Like-Biratnagar, herein tentatively named Feitosa virus (FEITV) (3 strains), CDBV (1 strain), NEGV (2 strains), WALV (2 strains). Many of these viruses have been detected in the same pool of hematophagous diptera (Table 2).

**Table 2:** Accession number of the isolate’s sequences deposited in the Genbank according to the mosquito specie and place of collection

Virus	Lab. record	Mosquito specie	Place of collection	Accession number
BRJV	BE AR 805511	*Cx. (Cux.) species*	Canaã dos Carajás, PA, BR	MZ615324
	BE AR 805514	*Cx. (Cux.) species*	Canaã dos Carajás, PA, BR	MZ615325
	BE AR 805520	*Cx. (Cux.) species*	Canaã dos Carajás, PA, BR	MZ615328
	BE AR 805525	*Cx. (Cux.) species*	Canaã dos Carajás, PA, BR	MZ615330
	BE AR 820396	*Cx. (Cux.) species*	Canaã dos Carajás, PA, BR	MZ615333
	BE AR 805503	*Cx. (Cux.) species*	Canaã dos Carajás, PA, BR	MZ615334
	BE AR 805503	*Cx. (Cux.) species*	Canaã dos Carajás, PA, BR	MZ615335
CDBV	BE AR 805503	*Cx. (Cux.) species*	Canaã dos Carajás, PA, BR	MZ615320
FEITV	BE AR 805503	*Cx. (Cux.) species*	Canaã dos Carajás, PA, BR	MZ615321
	BE AR 805514	*Cx. (Cux.) species*	Canaã dos Carajás, PA, BR	MZ615326
	BE AR 805529	*Cx. (Cux.) coronator*	Curionópolis, PA, BR	MZ615331
NEGV	BE AR 805503	*Cx. (Cux.) species*	Canaã dos Carajás, PA, BR	MZ615322
	BE AR 805514	*Cx. (Cux.) species*	Canaã dos Carajás, PA, BR	MZ615327
WALV	BE AR 805503	*Cx. (Cux.) species*	Canaã dos Carajás, PA, BR	MZ615323
	BE AR 805520	*Cx. (Cux.) species*	Canaã dos Carajás, PA, BR	MZ615329

Descriptive genome analysis shows that 5′ and 3′ non-coding regions (NCR) were found, which varied in size among the detected viruses from 17 to 562 nt and 143 nt to 534 nt, respectively. In addition, coding regions (ORFs) were also observed, with most viruses showing three ORFs (ORF 1, ORF 2 and ORF 3), except for CDBV, which showed a single ORF (ORF 1) 7023 nt in size. For the aforementioned viruses obtained, ORF 1 ranged in size from 6723 nt to 7107 nt, ORF 2 from 1203 nt to 1269 nt, and ORF 3 from 585 to 627 nt. As for the total nucleotide count of the samples, it ranged from 7574 nt to 9855 nt. Genome coverage ranged from 61.1 to 26,329 nt.

The protein domain analysis for ORF 1 of the isolated virus of the taxon *Negevirus* (FEITV, CDBV, WALV, BRJV and NEGV) performed through the InterProScan software using the databases (PFAM, PROSITE, PROFILE and PANTHER) showed the recognition of conserved domains for alphavirus MT, VMethyltransf, FtsJ, PSRV_Helicase (Viral Helicase 1), Ribosomal RNAm and RdRp. Regarding to viruses presenting ORF 2, the PFAM database identified only the protein domain for DiSB, solely in the BRJV and NEGV strains. With regard to ORF 3, the PFAM database recognized protein domain for SP24 in all strains of the isolated negevirus that have ORF 3 (FEITV, WALV, BRJV and NEGV).

**Fig. 2:**
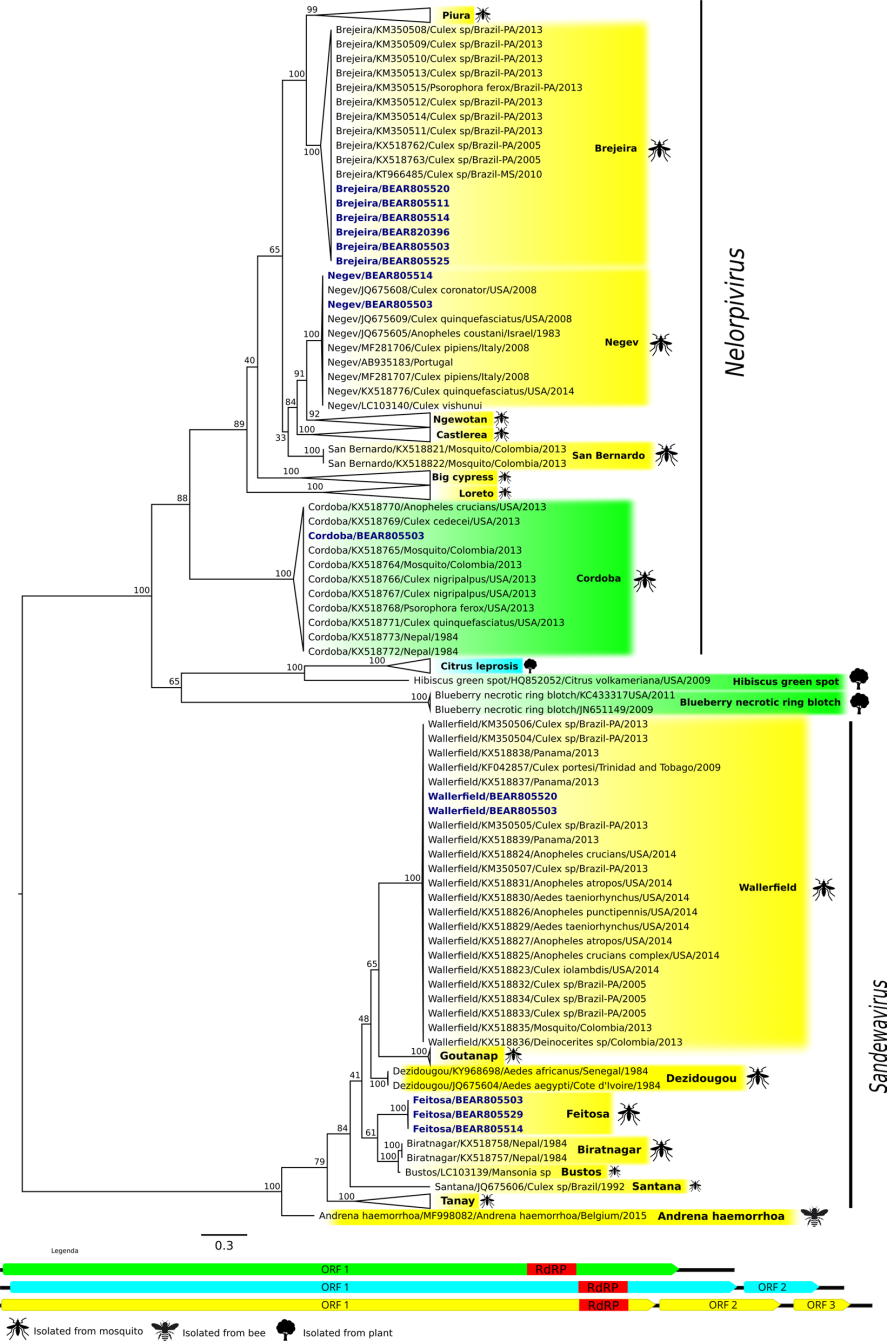
Phylogenetic tree based on the amino acid sequences of viral polymerase (RdRp) from ORF 1 of insect-specific viruses belonging to the taxon Negevirus. Phylogeny generated with the Maximum Likelihood method and LG model. The virus strains obtained in the study are in blue font and arranged in a cartoon format, while the virus groups related to the isolated viruses are collapsed. Viruses with all three ORFs are highlighted in yellow; those with two ORFs are highlighted in blue; and those with only one ORF are highlighted in green. The host that originated each isolate is identified with an image of the host (mosquito, bee, plant), identified in the legend. The bottom bar represents the rate of amino acid replacement

For phylogenetic analysis, a tree of the polymerase domain of ORF 1 of the sequenced negeviruses, BRJV, NEGV, CDBV, WALV and FEITV was first constructed, since all the viruses obtained have ORF 1. The isolated negeviruses clustered within the main groups previously described for these viruses: *Nelorpivirus* (BRJV, NEGV, CDBV) and *Sandewavirus* (WALV and FEITV). The isolated BRJV strains (BE AR 805520, BE AR 805511, BE AR 805514, BE AR 820396, BE AR 805503, and BE AR 805525) formed a group with the other previously described Brazilian BRJV strains, this group being more closely related to that of the PIUV. The NEGV strains obtained (BE AR 805514 and BE AR 805503) formed a single clade with the previously identified strains of this virus from the United States and Europe, with the NEGV group being more closely related to the NWTV. The CDBV strain, BE AR 805503, clustered with the other strains of this virus. The negeviruses of the *Nelorpivirus* genus have common ancestry with some plant viruses, such as the Citrus leprosis virus group C (CiLV-C), Hibiscus green spot virus (HGSV), and Blueberry necrotic ring blotch (BNRB). Regarding the negeviruses belonging to the *Sandewavirus* genus, the detected WALV strains—BE AR 805520 and BE AR 805503—formed a clade with the other American strains (Brazil, Panama, Trinidad and Tobago, USA and Colombia), with the WALV clade being more closely related to that of GANV. In turn, the FEITV relates more to the BIRV and BUSV (Fig. 2).

**Fig. 3:**
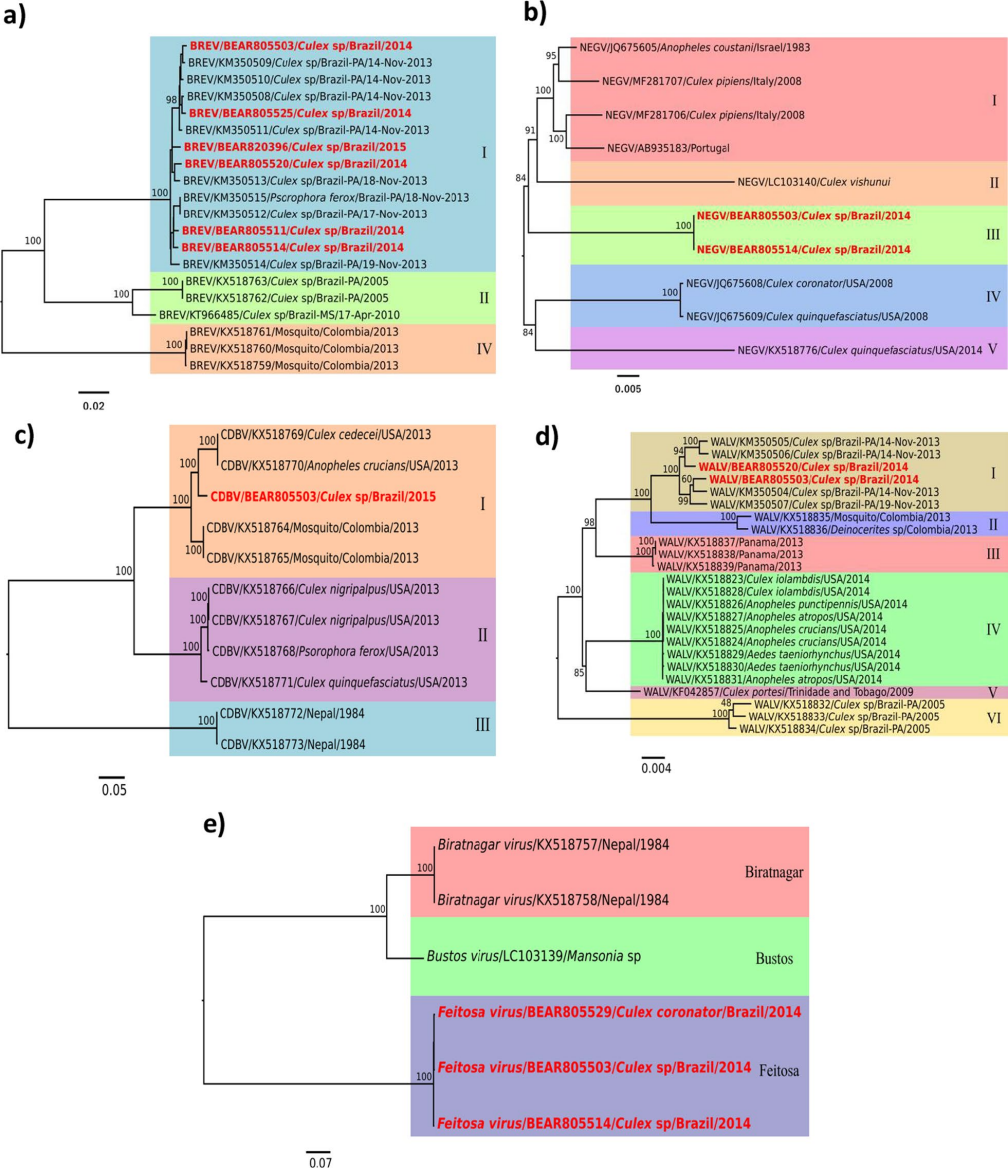
Phylogenetic tree of the concatenated nucleotide sequences of ORF1, ORF2 and ORF3 of the isolated strains (in red font) and other strains available in the Genbank, using the Maximum Likelihood method and GTR model. The bottom bar represents the rate of nucleotide replacement. **a** BRJV; **b** NEGV; **c** CDBV; **d** WALV; **e** FEITV

The phylogeny of BRJV showed the formation of three clades, group I corresponding to clades of Brazilian strains isolated in 2013, 2014 and 2015, including the strains isolated in the herein study. Group II is made up of isolates from Brazil in 2005 and 2010. On the other hand, group III is made up of the isolates from Colombia in 2013. All six BRJV strains that were isolated (Canaã dos Carajás and Curionópolis areas), in relation to the three concatenated ORFs, were shown to be more genetically related to the 2013 strains also from Canaã dos Carajás described by Nunes et al. [17], while the other Brazilian samples taken in 2005 and 2010 (group II) were shown to be more genetically distant from group I, with genetic distances ranging from 10.5 to 11.4% (Fig. 3a).

The isolated NEGV strains clustered in a different clade from that previously identified strains from the USA, Portugal, Italy, and Israel. Four groups were formed, differentiated by geographic region (Europe, Asia, Brazil, and USA, respectively). Group I was made up of the strains isolated in Israel, Italy, and Portugal; group II consisted only in the strain isolated in the Philippines; group III included the NEGV samples isolated in this study, and group IV and V included the viruses from the USA. Although the BE AR 805503 and BE AR 805514 strains are the most genetically distant from the other NEGV strains, with short nucleotide distances ranging from 4.2 to 6.6%, all strains from all groups, including the strains in this study, were shown to be strains of the same virus with nucleotide distances in a range of 0.1–6.6%. It is important to note that NEGV had not yet been isolated in Brazil (Fig. 3b).

The CDBV strain isolated from sample BE AR 805503, from *Culex species* mosquitoes collected in 2015, is the first isolation of this virus in Brazil. The Phylogenetic analysis of ORF 1 (the only identified ORF of this virus) showed the formation of three distinct clades (groups I, II, and III) involving strains isolated in several places around the world, such as the USA, Colombia, Brazil, and Nepal. The sample isolated hereby was shown to be related to strains isolated in Colombia and the United States in 2013, which formed group I, being more genetically distant from groups II and III viruses and made up by viruses detected in the United States and Nepal, respectively, with nucleotide distances ranging from 15.8 to 25.1% (Fig. 3c).

The WALV isolated from samples BE AR 805520 and BE AR 805503, from *Culex (Cux) species*, collected in 2014 in Canaã dos Carajás, were grouped in the same group as the strains identified in the previous year, 2013, also collected in Canaã dos Carajás area, which formed group I, being even more genetically distant, around 4–4.3%, in nucleotide terms from the strains isolated in 2005 (group VI) in the same state (Pará), but in a different city, called Trairão. Besides, other groups were formed based on geographic distribution, with groups II, III, IV and V consisting of viruses from Colombia, Panama, the USA and Trinidad and Tobago, respectively (Fig. 3d).

The FEITV, a new virus to science, was isolated in three out of the eight sequenced samples (BE AR 805529, BE AR 805503 and BE AR 805514), two of which from *Culex (Cux.) species* and one from *Culex coronator*, all collected in 2014 in Canaã de Carajás area. These samples grouped into a distinct clade, being most closely related to the BUSV and BIRV isolated in the Philippines and Nepal, respectively. Despite this comparison, it is a different virus, which showed a nucleotide genetic distance ranging from 39.3 to 39.6% (Fig. 3e).

The genomic sequences of the isolated strains were deposited at the Genbank database under the accession numbers informed in the Table 2.

## Discussion

This study aimed to investigate the circulation of arboviruses and ISVs from hematophagous arthropods collected near to areas of mining in the southeastern Pará state, Brazil. Investigations like this of entomosurveillance, which analyze how anthropological impacts could affect the natural balance of biodiversity in the region, are very important.

The sequenced ISVs strains showed CPE only in C6/36 cells. This result corroborates the study of Vasilakis et al. [7], which showed that six ISVs (NEGV, PIUV, DEZV, NWTV, LORV and SANV), caused CPE only in C6/36 cells and inoculated mice did not become ill, thus showing the replication restriction of ISVs only to mosquitoes and their cells.

Descriptive genome analysis of the viruses sequenced through this study shows that 5′ and 3′ non-coding regions (NCRs) and coding regions (ORFs) described for negeviruses were found according to Nunes et al. [8]. The protein domains recognized by the Interproscan software in ORF 1, ORF 2 and ORF 3 of the negeviruses isolated in this study were also recognized in studies performed by Vasilakis et al. [7] and Nunes et al. [8], who demonstrated the presence of these domains for most negeviruses (GANV, BIRV, DEZV, BREV, NEGV, PIUV, San Bernardo virus (SBNV), TANV, WALV, NWTV, SANV and BCPV), except the conserved domain for *Alphavirus* MT, found in ORF 1 and ORF 2 of WALV, which had not yet been described for negeviruses; also the conserved protein domain for DiSB was not recognized in the FEITV [8].

The phylogenetic analysis of the polymerase domain of ORF 1 of the detected negeviruses (BRJV, NEGV, CDBV, WALV and FEITV) showed that the strains obtained hereby clustered with the strains of these viruses and within groups (genera) previously established by Kallies et al. [3] and also described by Nunes et al. [8], *Nelorpivirus* (BRJV, NEGV, CDBV) and *Sandewavirus* (WALV and FEITV), highlighting the inclusion of a new negevirus obtained herein, FEITV, within the genus *Sandewavirus*. Furthermore, the arrangement of the virus groups in the phylogenetic tree and the genetic relationship between them agreed what was described by Nunes et al. [8].

BRJV, belonging to the genus *Nelorpivirus*, was isolated in Brazil in 2005 and 2013, in the North region, in the state of Pará; it was also isolated in Colombia in the Cordoba region in 2013, and in Mato Grosso do Sul state (Pantanal) in 2010. In most studies, the virus was isolated from *Culex sp.* [25]; this study also isolated six strains of BRJV from a pool of *Culex (Cux.) species* collected in the Canaã de Carajás and Curionópolis areas in 2014 and 2015, respectivamente.

NEGV is part of the *Nelorpivirus* genus and has been isolated from several mosquito species such as *Culex quinquefaciatus, Culex univitatus, Anopheles constant, Culex coronator,* and *Ochlerotatus caspius.* This virus has been identified in the USA, Portugal, Italy, and Israel [6–9]. In the herein study, two strains of the NEGV were identified, and this is the first description of the virus in Brazil, specifically in the state of Pará. The strains were also isolated from arthropods of the *Culex (Cux.) species* collected in the Canaã dos Carajás area in 2014.

The first isolation of CDBV in Brazil was obtained in our study, from *Culex (Cux.) species* collected in 2015. Phylogenetic analysis of ORF 1 showed the formation of three distinct clades (groups I, II and III), whereas, Nunes et al. [8] described the formation of two groups, group I including the same strains used herein from Colombia and the USA in 2013 and group II, including viruses from Nepal.

WALV has already been isolated in several regions in the world such as Brazil, Trinidad and Tobago [4], the USA, Colombia and Panama between 2005 and 2014. These samples were isolated from several species of hematophagous diptera, such as *Deinocerites sp., Anopheles atropos, Anopheles punctipennis, Anopheles crucians, Culex iolambdis, Anopheles crucians, Aedes taeniorhynchus, Culex sp. and Culex declarator.* We isolated WALV strains from *Culex* species mosquitoes which were more genetically related to strains also from Brazil.

This study isolated a new negevirus for science, tentatively named FEITV, related to negeviruses of the genus *Sandewavirus*, BIRV and BUSV, due to the fact that it was obtained from hematophagous diptera captured in Vila Feitosa area, in Canaã dos Carajás municipality, in 2014. Despite being most closely related to these two viruses, the three FEITV strains that were isolated grouped into a distinct clade and showed nucleotide difference ranging from 39.3 to 39.6%, thus showing that it is a different virus and a new member of the taxon *Negevirus*. In fact, FEITV demonstrated to have genomic organization compatible with the organization of negeviruses of the *Sandewavirus* genus, with the presence of the three ORFs with sizes similar to those described for the other viruses of the taxon, and the recognition of conserved protein domains was observed in this virus as well as in the other negeviruses.

The importance of ISVs and their relationship with arboviruses has being investigated, for example the *Culex* mosquitoes infected by the insect-specific flavivirus, Culex flavivirus (CxFV), were less susceptible to secondary infection by West Nile virus (WNV). Further studies indicated that other insect-specific flavivirus, Nhumirim virus (NHUV), significantly reduced the replication of arboviruses such as WNV, Japanese Encephalitis virus (JEV) and Saint Louis Encephalitis virus (SLEV) in co-infected C6/36 cells. [26] Previous studies are also verifying the antiviral potential of other insect-specific flaviviruses, such as the Parramatta River virus (PaRV) [27]. Recently, a study conducted by Patterson et al. [28] demonstrated that certain negeviruses reduce alphavirus replication during in vitro co-infection.

Our results demonstrated the phylogenetic relationship between negeviruses and certain plant viruses, reinforce the necessity of further studies including analysis by molecular clock to better understand the aspects related to the evolution of both group of viruses. Hypotheses are raised as to evolutionary relationship between ISVs and plant viruses, standing out the ISVs of the taxon *Negevirus* and those of the family *Tymoviridae*. It is possible that because of the insect food habit of feeding on plant nectar and plant aquatic material (initial life cycles) [2], plant viruses may have evolved to become ISVs, passing to infect insects; otherwise, it is also possible that ISVs evolve to become plant viruses, which in turn can now infect plants [1]**.**

In the herein study it was not possible to evaluate the relationship between ISVs and arboviruses, but it is important to emphasize the importance of conducting studies that seek to evaluate their relationship with arboviruses in vitro and in vivo, even as potential control strategies for arboviruses, which is one of the possible applications of ISVs [1]. Furthermore, ISVs are still being studied with regard to the possibility of being used as platforms for safe diagnostic and vaccine development [1].

## Conclusions

Therefore, the study ascertained the occurrence of a variety of ISVs of the taxon *Negevirus* (BRJV, NEGV, CDBV, WALV and FEITV) in the Canaã dos Carajás area, in Pará state, Brazil. The BRJV has also been detected in Curionopolis, Pará state. It should be noted that two negeviruses were isolated for the first time in Brazil, namely the NEGV and the CDBV. C6/36 cells have proven to be a good system for isolation of ISVs, especially those of the taxon *Negevirus*. In the Canaã dos Carajás area, in Pará state, a new virus was detected for science and tentatively named Feitosa negevirus. No arboviruses of the *Flavivirus*, *Alphavirus, Orthobunyavirus* and *Phlebovirus* genera were detected in this study.

## Data Availability

The datasets used and analyzed during the current study are available from the corresponding author on reasonable request.

## Abbreviations

ISVs: Insect-specific viruses
dpi: Days post infection
CPE: Cytopathic effect
BRJV: Brejeira virus
CDBV: Corboda virus
LORV: Loreto virus
NEGV: Negev virus
NWTV: Ngewotan virus
PIUV: Piura virus
SBDV: San Bernardo virus
BIRV: Biratnagar virus
DEZV: Dezidougou virus
GANV: Goutanap virus
SANV: Santana virus
WALV: Wallerfield virus
BUSV: Bustos virus
CHIKV: Chikungunya virus
DENV: Dengue virus
OROV: Oropouche virus
